# Editorial: Synovial pathobiology and pathogenesis of inflammatory arthritis

**DOI:** 10.3389/fmed.2022.1008562

**Published:** 2022-08-16

**Authors:** Annabelle Small, Frances Humby, Mihir D. Wechalekar

**Affiliations:** ^1^College of Medicine and Public Health, Flinders University, Adelaide, SA, Australia; ^2^Department of Rheumatology, Guy's and St Thomas' NHS Foundation Trust, London, United Kingdom; ^3^Department of Rheumatology, Flinders Medical Centre, Adelaide, SA, Australia

**Keywords:** synovium, rheumatoid arthritis, pathogenesis, inflammatory arthritis, precision medicine

The current holy grail for clinicians and researchers investigating inflammatory arthritis (IA), including rheumatoid arthritis (RA), are tools that will allow accurate prediction of the disease course in an individual patient. Ideally, these tools will allow prediction at three critical stages: (i) in undifferentiated IA, prior to progression to definite diagnosis, (ii) prognosis following diagnosis, and (iii) prediction of response to therapeutics. The collection of original research and review articles published in the topic *Synovial pathobiology and pathogenesis of inflammatory arthritis* neatly review and provide insights into current endeavors to find these tools, and future strategies that may be adopted to make these much-needed predictions.

Remission in RA, probably best conceptualized as being at the very end of a continuum of disease activity, and currently measured by any of the various composite disease activity scores, remains the goal for patients with RA and the rheumatologists treating them. However, despite advances in current therapeutics, rates of sustained remission remain as low as ~19% (1). This indicates that there remains a pressing need for greater insights into the process of reaching remission, defining objective markers of remission, and the prediction of the patients for whom this is achievable. In their mini review, Bugatti et al. discuss definitions of remission of RA synovitis and the signs of “deep” remission at the clinical, imaging and pathological level. There is currently a lack of standardization in the definition of clinical remission and summarizing each currently used method of assessing disease activity and remission highlights their unique advantages and limitations, making it plain that complimentary analytical tools are needed to facilitate the combination of these perspectives and improve remission evaluation. The authors summarize by posing the thoughtful question of whether the observation of a relatively small proportion of RA patients achieving sustained, drug-free remission suggests a long-term possibility that RA synovitis may be fully and stably suppressed.

In this topic, imaging prediction models, specifically ultrasonographic examination, are a recurring theme. Such models are an attractive choice for informing prognosis due to their cost-effectiveness, non-invasiveness, and speed. The contribution from Humby et al. succinctly summarizes efforts at establishing predictive models for RA diagnosis, prognosis, and response to biologic treatments, which combine clinical parameters and joint imaging. However, while there have been advances in this space, currently available clinical models remain insufficient for predicting early diagnosis and prognoses. Complimenting clinical and imaging models, is the pathology of the target tissue of RA, the synovial tissue (ST). Finally, the authors discuss the integration of ST pathobiology with current clinical and imaging prediction models. While the high degree of heterogeneity seen in the RA ST poses an enormous challenge to marrying ST pathobiology with prediction models, it is almost certain that with a personalized approach, as utilized in the treatment of cancer, this combination will provide the path forward to improving prediction models for clinical outcomes of patients with RA.

Of course, while clinical parameters and peripheral blood biomarkers are the “gold standard” for a predictive tool of RA pathology, these have thus far had limited success in accurately predicting individual disease. Thus, studies akin to those discussed by Humby et al. into using the pathobiology of the ST to generate predictive tools are becoming more prevalent. Neatly complementing the above works, is the original research by Alivernini et al., who examined the target tissue of IA, the ST, for predictors of progression from seronegative undifferentiated peripheral IA into definite seronegative arthritis. With a combination of Gray Scale and power Doppler evaluation and ultrasound guided biopsy, the authors demonstrate correlation between microRNA signature, histological and ultrasound features of ST with the differentiation into seronegative arthritis, concluding that these parameters together may accurately assist in the identification of patients whose disease will go on to develop seronegative arthritis.

The development of high throughput technologies has provided tools that allow our search for potential biomarkers and therapeutic targets in the ST to be conducted in more depth than has previously been possible. Using RNA sequencing, Zhang et al. investigated the transcription profiles of the ST in a cohort of 9 RA patients undergoing total knee replacement, compared with 15 osteoarthritis (OA) patients using a bulk transcriptomic approach. The authors found significant differences between the RA and OA synovial transcriptome, with 851 differentially expressed genes identified (474 upregulated, 377 downregulated), and suggest the quantitation of specific hub genes as a predictive tool. Specifically, the study revealed differential expression of 22 genes encoding chemokines and chemokine receptors, and accordingly, Kyoto Encyclopedia of Genes and Genomes (KEGG) pathway analysis of identified differentially expressed genes revealed that these were enriched in cytokine-cytokine receptor interaction, chemokine signaling, and T cell receptor signaling. These findings highlight the role of immune pathways and chemokine signaling in RA pathogenesis, which may serve as potential diagnostic and therapeutic targets in the future.

In terms of newer therapeutic targets for RA, a current prominent target is the Janus kinase (JAK)-Signal transducer and activator of transcription (STAT) pathway. As a central signaling pathway in the human immune response, this pathway poses an attractive target for the treatment of immune-mediated disorders, and the first JAK inhibitor was approved for the treatment of RA in 2012. Since then, this pathway and its inhibition have been extensively studied in RA. In their contribution, Burja et al. review the effects of the FDA-approved JAK inhibitors for inflammatory arthritis (Tofacitinib, Baricitinib, Upadacitinib, Peficitinib) on joint damage and synovitis in RA as found in four recent clinical trials. Together, these demonstrate that tofacitinib either as a monotherapy or in combination with methotrexate decreased progression of joint damage in RA, and that this effect was transcriptional rather than cellular. Next, the authors focus on the effects of JAK inhibitors on synovial fibroblast-mediated pathology, where they detail the known effects of JAK inhibitors on fibroblast biology *in vitro*, draw light to the limitations of *in vitro* experimental outcomes vs. the outcomes observed *in vivo*, and finally summarize the activities of JAK inhibitors that remain to be explored.

As a whole, the topic *Synovial pathobiology and pathogenesis of inflammatory arthritis* details the research questions of reaching remission in RA, summarizes approaches by which we may better predict the course of IA and RA and whether remission presents an attainable goal, and captures complexities in IA research. While the endeavor of finding accurate predictive tools for IA remains a major challenge and unmet need, the concerted global efforts of researchers summarized within this topic provide insights and hope into accurate predictive tools and precision medicine in this field.

## Author contributions

All authors listed have made a substantial, direct, and intellectual contribution to the work and approved it for publication.

## Funding

We acknowledge that AS was supported by funding from an Ideas Grant from the National Health and Medical Research Council (NHMRC) of Australia (Grant Number APP2004839).

## Conflict of interest

The authors declare that the research was conducted in the absence of any commercial or financial relationships that could be construed as a potential conflict of interest.

## Publisher's note

All claims expressed in this article are solely those of the authors and do not necessarily represent those of their affiliated organizations, or those of the publisher, the editors and the reviewers. Any product that may be evaluated in this article, or claim that may be made by its manufacturer, is not guaranteed or endorsed by the publisher.
